# Development and validation of nomograms for predicting grade ≥3 diarrhea and neutropenia after abemaciclib combined with endocrine therapy for breast cancer: a multicenter retrospective real-world study

**DOI:** 10.3389/fonc.2025.1515420

**Published:** 2025-10-06

**Authors:** Lei Wang, Siyuan Yang, Ji Zhang, Hairui Wang, Ying Zhang, Xin Wang, Meng Shen, Chunmei Ye, Taiwen Deng, Yujin Ying, Yang Li, Jianyun Nie

**Affiliations:** ^1^ Department of Breast Surgery, The Third Affiliated Hospital of Kunming Medical University, Yunnan Cancer Hospital, Kunming, Yunnan, China; ^2^ Department of General Surgery , No. 926 Hospital of the Joint Logistic Support Force of the Chinese People's Liberation Army, Kaiyuan, Yunnan, China; ^3^ Department of Thyroid and Breast Surgery, The Third People’s Hospital of Yunnan Province, Kunming, Yunnan, China; ^4^ Department of Breast Surgery, The First Affiliated Hospital of Kunming Medical University, Kunming, Yunnan, China; ^5^ Department of Gastroenterology and Oncology, The Third Affiliated Hospital of Kunming Medical University, Yunnan Cancer Hospital, Kunming, Yunnan, China

**Keywords:** breast cancer, CDK4/6 inhibitors, abemaciclib, adverse events, risk factor, real-world

## Abstract

**Background:**

This study aimed to investigate the risk factors associated with grade ≥3 diarrhea and neutropenia, which are the most common adverse events (AEs) leading to discontinuation and dose reduction in patients with hormone receptor-positive (HR+)/human epidermal growth factor 2-negative (HER2-) breast cancer treated with cyclin-dependent kinase 4/6 (CDK4/6) inhibitor abemaciclib combined with endocrine therapy (ET). Subsequently, two prediction nomograms were developed to serve as a foundation for enhancing the management of patients’ side effects and improving treatment quality.

**Methods:**

A retrospective cohort analysis was conducted to explore the clinical characteristics and treatment variables of breast cancer patients treated with abemaciclib combined with ET in Yunnan Cancer Hospital from December 2021 to December 2022. Logistic regression was used to determine the risk factors for the occurrence of grade ≥3 diarrhea and neutropenia, and two kinds of nomograms were established. An external validation group of patients from three additional centers was used to validate the constructed nomograms. The area under the receiver operating characteristic (ROC) curve (AUC), calibration curve, and decision curve analysis (DCA) were used to assess the predictive performance and clinical applicability of the two nomograms.

**Results:**

A total of 497 patients were included, including 403 in the modeling group and 94 in the external validation group. The results of the multifactorial analysis revealed that age ≥70 years, Eastern Cooperative Oncology Group (ECOG) score ≥1, and underlying gastrointestinal diseases were independent risk factors for grade ≥3 diarrhea. ECOG score ≥1, radiotherapy in the same period/within 1 month, and neutrophils ≤2.0×10^9^/L before treatment were independent risk factors for grade ≥3 neutropenia. Two nomogram models were used to predict risk based on the above independent factors. The AUCs for the developmental and external validation groups were 0.747(95%CI:0.687-0.806) and 0.803(95%CI:0.702-0.918) for the diarrhea prediction nomogram and 0.765(95%CI:0.711-0.818) and 0.783(95%CI:0.691-0.892) for the neutropenia prediction nomogram, respectively. Calibration curves and DCA of both models also showed good predictive performance and clinical applicability.

**Conclusion:**

We identified risk factors for grade ≥3 diarrhea and neutropenia in patients treated with abemaciclib combined with ET, and established a risk prediction nomogram, providing a scientific basis for safety assessment.

## Introduction

Breast cancer is the number one health threat to women worldwide. Hormone receptor-positive (HR+)/human epidermal growth factor 2 negative (HER2–) breast cancer constitutes 60–70% of all breast cancer cases (1). For patients with HR+/HER2 metastatic or locally advanced breast cancer, as well as for patients with early breast cancer with specific risk factors, the use of CDK4/6 inhibitors combined with endocrine therapy (ET) has become the primary recommended regimen to significantly reduce the risk of disease progression or mortality (2–4). However, studies have shown that approximately 14.5–25.1% of patients discontinue treatment due to intolerance to severe adverse events (AEs) (5), which has a significant impact on patients’ ability to enjoy their quality of life and adhere to treatment. Nevertheless, the occurrence of AEs associated with drug therapy and the incidence and severity of these effects vary considerably (6, 7). Consequently, it is of paramount importance to accurately identify the risk factors for AEs following the use of CDK4/6 inhibitors and implement effective coping strategies in advance, with the objective of reducing the risk of AEs and optimizing the treatment effect and quality of life of patients.

Abemaciclib is a widely used oral CDK4/6 inhibitor in clinical practice. Current research shows that the most common AEs caused by dosage reduction or cessation in patients treated with abemaciclib are diarrhea and neutropenia (8, 9). Clinical studies have also shown that advanced age, menopause, and gastrointestinal diseases may increase the risk of diarrhea in patients with abemaciclib (10–12), and that neutropenia may be related to race, Eastern Cooperative Oncology Group (ECOG) score, and white blood cell level before treatment (13). Nevertheless, the enrollment of patients in large-scale clinical trials is strictly limited, precluding the ability to fully reflect the real-world occurrence of AEs. Currently, there is a lack of detailed studies on the risk factors related to the occurrence of AEs following the use of abemaciclib, a lack of systematic evaluation of the risk factors related to the occurrence of AEs in high-risk populations, and a lack of effective predictive models for intuitive risk assessment.

This study aimed to identify the risk factors associated with grade ≥3 diarrhea and neutropenia in patients with HR+/HER2- breast cancer treated with abemaciclib in combination with ET in a real-world setting. Furthermore, this study aimed to construct two risk prediction nomogram models to graphically represent the regression equation. The efficacy of the models was assessed through internal and external validations with the aim of developing a validated predictive tool for the individualized management of AEs.

## Materials and methods

### Study population

The clinical characteristics and therapeutic variables of patients with breast cancer treated with abemaciclib and ET were retrospectively collected at Yunnan Cancer Hospital, First Affiliated Hospital of Kunming Medical University, First People’s Hospital of Kunming and Third People’s Hospital of Yunnan Province from December 2021 to December 2022. Following the application of rigorous inclusion and exclusion criteria, 269 patients from the Yunnan Cancer Hospital and 94 patients from three additional hospitals were selected as the development and external validation groups, respectively. The following criteria were included (1): females aged ≥18 years (2); pathologically confirmed HR+/HER2– breast cancer (3); at least one cycle of standard-dose CDK4/6 inhibitor combined with ET; and (4) patient and family consent. The following were excluded (1): visceral crisis, other serious diseases, or other malignant tumors (2); inflammatory breast cancer (3); no assessment of adverse drug reactions and patients lost to follow-up; and (4) lack of baseline clinicopathological and hematological data. This study was approved by the Ethics Committee of the Third Affiliated Hospital of Kunming Medical University, Yunnan Cancer Hospital (approval number: KYLX2023-011).

### Data collection

The following clinical data was obtained: age, body mass index (BMI), ECOG score, menopausal status, pathological information (clinical stage, molecular typing), types of combined ET (aromatase inhibitors (AI), fulvestrant), combined with other drugs (anti-bone metastasis therapy, chronic disease drugs, etc.), body surface area (BSA), metastasis status (quantity and position), previous antineoplastic therapy (chemotherapy, radiotherapy, ET), antibiotic use during treatment, combined underlying diseases, and hematological parameters before treatment. Underlying diseases included cardiovascular diseases (coronary heart disease, heart disease and hypertension), metabolic diseases (diabetes, hyperlipidemia, hyperuricemia, hyperthyroidism, and hypothyroidism), gastrointestinal diseases (colitis, irritable bowel syndrome, inflammatory bowel disease, constipation, chronic diarrhea, food intolerance, and pelvic radiotherapy), and a history of liver diseases (hepatitis, cirrhosis, and fatty liver). Hematological parameters before treatment included neutrophil (NE) count, white blood cell (WBC) count, albumin (propagated), aspartate aminotransferase (AST), alanine aminotransferase (ALT), alkaline phosphatase (ALP), and serum creatinine (Cr) levels. The hematological data were obtained from the most recent hematological examination conducted 21 days before abemaciclib administration.

### Drug use methods

The patients in this study received abemaciclib at an initial dose of 150 mg bis in die (BID), in combination with AI, such as anastrozole, letrozole, and exemestane, or in combination with selective estrogen receptor downregulatory (SERD) fulvestrant. Dose adjustments were made during patient administration based on safety and tolerability. The first recommended dose adjustment was 100 mg BID.

### Follow-up

Patients were followed-up by telephone and case review for 12 months following drug administration or until they developed intolerable AEs and discontinuation was caused by disease progression. During the follow-up period, all AEs occurring in patients were recorded; if multiple AEs occurred, the highest-grade AE was recorded. The seriousness of the AEs was graded according to the Criteria for the Evaluation of Therapeutic Adverse Events (CTCAE version v5.0) (14).

### Statistical analysis and model construction

**Figure 1:**
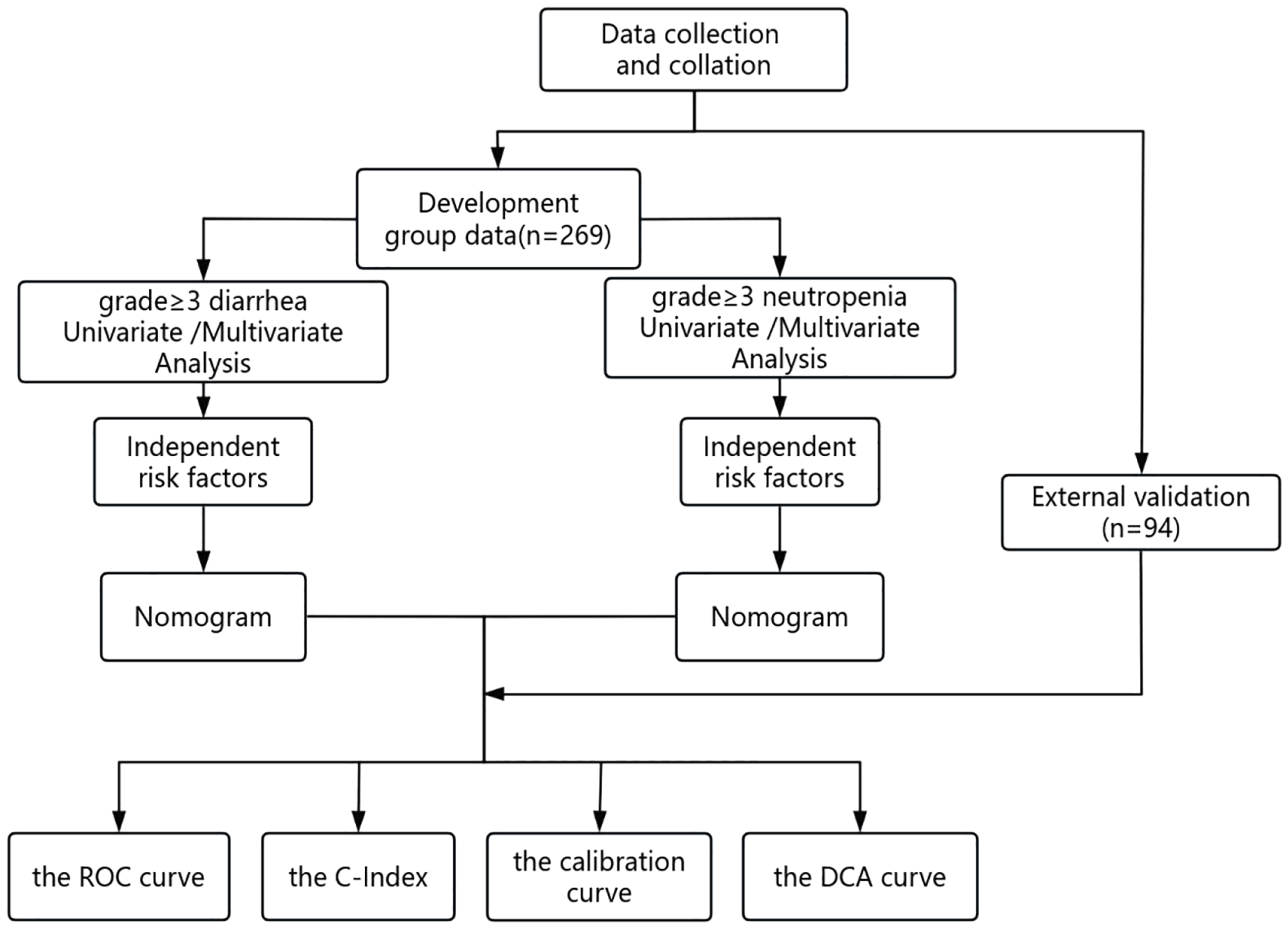
Flowchart for the methods.

Statistical analysis was conducted using SPSS 26.0 (IBM Corp., Armonk, NY, USA). Descriptive statistics were used to summarize measures, including averages with standard deviations, counts, or scores based on clinical cutoffs. Frequencies and percentages were used to describe nominal variables. Group comparisons were made using independent samples t-tests for measures and Pearson chi-squared or Fisher’s exact tests for counts. Univariate analyses evaluated all clinical characteristics and treatment variables associated with AEs. To ascertain the independent associations between the variables and AEs, those with P < 0.1 in the univariate analysis were entered into a multivariate logistic regression. Independent risk factors identified from the multifactorial analysis were used to construct a nomogram model using R software version 4.3.1 (Foundation for Statistical Computing, Vienna, Austria). Model discriminative ability was assessed using C-index and area under the receiver operating characteristic (ROC) curve (AUC), with internal validation using 1000 bootstrap resampling. Calibration curves assessed model accuracy, and clinical utility was assessed using decision curve analysis (DCA). External validation was performed using comparable differentiation, calibration, and clinical utility assessments. Statistical significance was set at α = 0.05. **Figure 1** illustrates the study methodology flowchart.

## Results

### Real-world occurrence of AEs

In total, 363 patients treated with abemaciclib combined with ET were included. During the follow-up period, approximately 98% of patients developed AEs of varying severity. A total of 27 AEs were monitored, and a total of 23 AEs with an incidence ≥10% were observed (**Supplementary Table S1**). The three most prevalent grade ≥3 AEs were neutropenia (27.4%), diarrhea (19.9%), and fatigue (10.3%). AEs that resulted in treatment discontinuation were observed in 115 patients (23.4%), which included 38 patients (7.6%) with diarrhea and 28 patients (5.6%) with neutropenia. AEs resulting in dose reduction were observed in 91 patients (18.3%), which included 31 patients (6.2%) with diarrhea and 15 patients (3.0%) with neutropenia.

In the therapeutic regimen of abemaciclib combined with Endocrine Therapy (ET), the specific ET agents administered were as follows: fulvestrant in 210 cases (42.3%), exemestane in 47 cases (9.5%), anastrozole in 182 cases (36.6%), and letrozole in 58 cases (11.7%). Among the AEs leading to the highest incidence of drug withdrawal and dose reduction, the incidence of diarrhea and neutropenia of any grade and grade ≥3 did not differ significantly between the groups combined with different ET treatments (**Supplementary Table S2**).

### Baseline characteristics for development and external validation groups

The study included 363 patients treated with abemaciclib plus ET, with 403 in the developmental group and 94 in the external validation group. The average age of the development group was 53.05 ± 11.14 years, and that of the external validation group was 51.12 ± 12.74 years. After all patients were followed up, it was found that 79 patients had ≥ grade 3 diarrhea in the modeling group, with an incidence of 19.6%, and 107 patients had ≥ grade 3 neutropenia, with an incidence of 26.6%. In the external validation group, 20 patients had grade ≥3 diarrhea, with an incidence of 21.3%, and 29 patients had grade ≥3 neutropenia, with an incidence of 30.9%. Baseline characteristics were not significantly different between the two groups, and general clinical information is shown in **Table 1**.

**Table 1:** Baseline characteristics of the development group and external validation group.

Project	Total	Development group		External validation group		*P*
	N=497(%)	n=403	(%)	n=94	(%)	
Age						0.425
≥70	57(11.5%)	44	10.9%	13	13.8%	
<70	440(88.5%)	359	89.1%	81	86.2%	
Clinical stage						0.059
II	65(13.1%)	60	14.9%	7	5.3%	
III	151(30.4%)	126	31.3%	25	26.6%	
IV	281(56.5%)	217	53.8%	62	68.1%	
Molecular typing						0.278
Luminal A	308(62.0%)	237	58.8%	61	75.5%	
Luminal B	189(38.0%)	166	41.2%	33	24.4%	
ECOG						0.661
≥3	32(6.4%)	24	6.0%	8	8.5%	
1-2	178(35.8%)	145	36.0%	33	35.1%	
0	287(57.8%)	234	58.1%	53	56.4%	
BMI						0.329
≥25kg/m^2^	169(34.0%)	133	33.0%	36	38.3%	
<25kg/m^2^	328(66.0%)	270	67.0%	58	61.7%	
Menstrual status						0.230
After menopause	339(68.2%)	270	67.0%	69	73.4%	
Premenopausal/ perimenopausal period	158(31.8%)	133	33.0%	25	26.6%	
Number of Metastasis						0.169
0	226(45.5%)	189	46.9%	37	60.6%	
1-2	233(46.9%)	187	46.4%	46	48.9%	
≥3	38(7.6%)	27	6.7%	11	11.7%	
Endocrine combination treatment						0.447
AI	287(57.7%)	236	58.6%	51	54.3%	
Fulvestrant	210(42.3%)	167	41.4%	43	45.7%	
Underlying disease						0.567
yes	198(35.8%)	163	40.4%	35	37.2%	
no	299(74.2%)	240	59.6%	59	62.8%	
Previous chemotherapy						0.830
yes	441(88.7%)	357	88.6%	84	89.3%	
no	56(11.3%)	46	11.4%	10	10.6%	
Previous Radiotherapy						0.070
yes	281(56.5%)	220	54.6%	61	64.8%	
no	216(43.5%)	183	45.4%	33	35.1%	
Previous endocrine therapy						0.063
yes	211(42.5%)	163	40.4%	48	51.06%	
no	285(57.5%)	239	59.3%	46	48.94%	

BMI, body mass index.

### Risk factor analysis and nomogram construction for grade ≥3 diarrhea

#### Univariate and multivariate analysis of grade ≥3 diarrhea

To determine the independent risk factors for the occurrence of grade ≥3 diarrhea with abemaciclib and ET, risk factors were analyzed using univariate and multivariate logistic regression, as shown in **Table 2**. From the results of univariate analysis, grade ≥3 diarrhea was associated with age, ECOG, combined with underlying gastrointestinal disease, and radiotherapy in the same period/within 1 month*(p* < 0.05). To mitigate the effects of confounding factors, a multivariate logistic regression analysis was conducted, and it incorporated variables with p < 0.1 in the univariate analysis. The results showed that age ≥70 years, ECOG score ≥1, and combined with underlying gastrointestinal diseases were independent risk factors for grade ≥3 diarrhea following abemaciclib combined with ET.

**Table 2:** Univariate and multivariate analysis of grade ≥3 diarrhea.

Variables		Univariate analysis		Multivariate analysis	
		OR (95%CI)	*P* value	OR (95%CI)	*P* value
Age	<70	Reference			
	≥70	3.016(1.550,5.869)	0.001	3.228(1.521,6.850)	0.002
Molecular typing	II	Reference			
	III	1.196(0.561,2.548)	0.643		
	IV	0.849(0.412,1.750)	0.658		
ECOG	0	Reference			
	1-2	1.971(1.167,3.327)	0.011	2.552(1.424,4.572)	0.002
	≥3	3.547(1.438,8.749)	0.006	5.395(2.039,14.274)	0.001
Molecular typing	Luminal A	Reference			
	Luminal B	1.182(0.713,1.958)	0.517		
BMI	<25kg/m^2^	Reference			
	≥25kg/m^2^	0.583(0.332,1.025)	0.061	0.563(0.307,1.031)	0.063
Menstrual status	Premenopausal/ perimenopausal period	Reference			
	After menopause	0.936(0.557,1.575)	0.804		
Number of Metastasis	0	Reference			
	1-2	1.246(0.493,3.152)	0.642		
	≥3	0.699(0.416,1.173)	0.175		
Anti-bone metastasis	no	Reference			
	Diphosphonate	0.578(0.288,1.162)	0.124		
	Denosumab	0.484(0.197,1.190)	0.114		
Previous treatments	no	Reference			
	Previous endocrine therapy	1.065(0.646,1.755)	0.805		
	Previous chemotherapy	0.863(0.408,1.823)	0.863		
	Previous Radiotherapy	1.127(0.686,1.851)	0.637		
Underlying disease	no	Reference			
	Metabolic diseases	1.482(0.748,2.939)	0.259		
	Cardiovascular diseases	1.503(0.812,2.781)	0.195		
	Underlying liver disease	1.191(0.521,2.724)	0.679		
	Underlying gastrointestinal diseases	4.588(2.685,7.838)	<0.001	4.457(2.517,7.893)	<0.001
Endocrine combination	Fulvestran	Reference			
	AI	1.490(0.806,2.754)	0.204		
Combined with other drugs	0	Reference			
	1	1.307(0.789,2.164)	0.298		
	≥2	0.915(0.253,3.310)	0.892		
Antibiotic use > 7 days	no	Reference			
	yes	1.446(0.746,2.804)	0.275		
Simultaneous or combined radiotherapy within 1 month	no	Reference			
	yes	1.771(1.012,3.099)	0.045	1.509(0.814,2.797)	0.192
BSA		1.004(0.990,1.019)	0.591		
Hematology before treatment	WBC	0.997(0.910,1.093)	0.949		
	NE	0.966(0.858,1.087)	0.564		
	ALB	1.009(0.961,1.060)	0.719		
	ALP	0.999(0.994,1.004)	0.688		
	Cr	0.994(0.979,1.009)	0.437		
	ALT	1.000(0.981,1.018)	0.959		
	AST	0.990(0.968,1.012)	0.374		

BMI, body mass index; ET, endocrine therapy; BSA, body surface area; WBC, white blood cell count; NE, neutrophil count; ALB, albumin; ALP, alkaline phosphatase; ALT, alanine aminotransferase; AST, aspartate aminotransferase; Cr, serum creatinine.

#### Development and validation of a nomogram for grade ≥3 diarrhea

**Figure 2:**
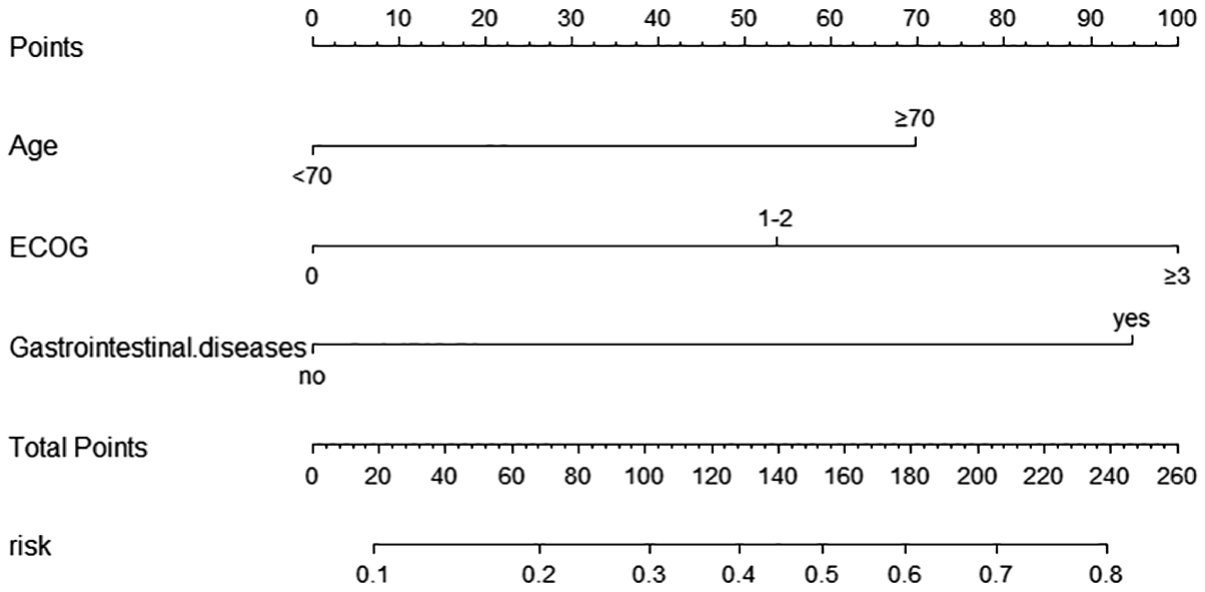
Nomogram for predicting the occurrence of ≥ grade 3 diarrhea in the modeling Group. Scores of risk factors: “Age” = Age, <70 = 0 points, ≥70 = 70 points; “ECOG” = ECOG score, 0 = 0points, 1-2 = 54 points, ≥3 = 100 points; “Gastrointestinal.disease” = complicated with underlying Gastrointestinal diseases, no=0 points, yes=95 points.

According to the multifactorial logistic regression analysis of independent risk factors for grade ≥3 diarrhea, a nomogram model was constructed using the “rms” package in R software, as shown in **Figure 2**. The factors in the nomogram were assigned points according to their effect on the dependent variable, and the point value line represents the estimated point value for each of the risk factors. The scores for each factor were summed to obtain the total score, and the frequency of grade ≥3 diarrhea was estimated from the total score. A higher score corresponded to a higher likelihood.

**Figure 3:**
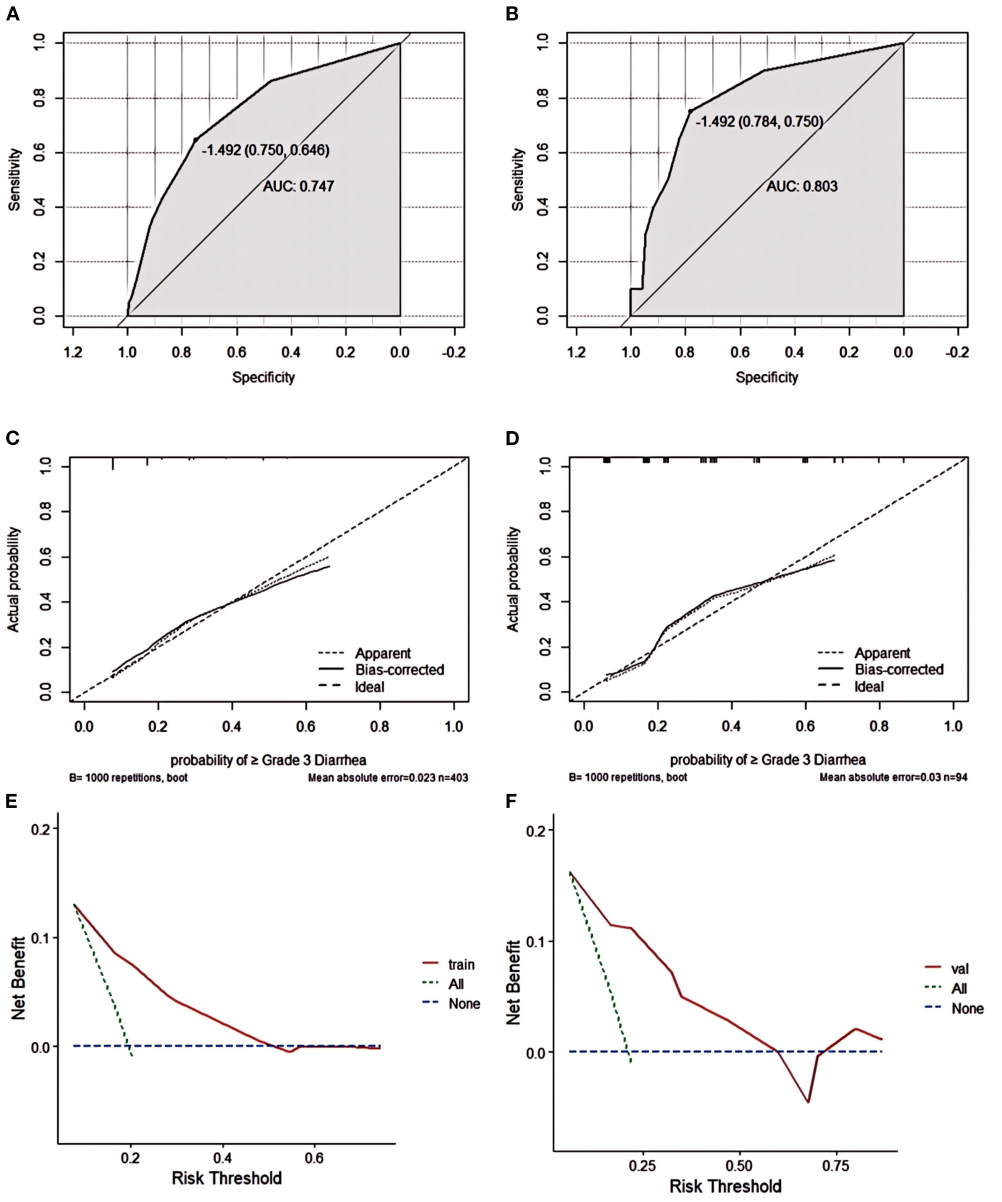
Grade ≥3 diarrhea nomogram evaluation **(A–F)**. ROC curves of the nomogram for the training set **(A)** and the external validation set **(B)**. The AUC values of the training cohort and the external validation cohort were 0.747 and 0.803, respectively.Nomogram calibration curves for training set **(C)** and validation set **(D)**. DCA curves for the training set **(E)** and validation set **(F)**.

Nomogram model performance was evaluated by C-index calculation and plotting ROC, calibration, and DCA curves as shown in **Figure 3**. Internal validation of the model showed a C-index of 0.747, and the ROC curve results showed an AUC of 0.747(95% CI: 0.681–0.829), a sensitivity of 0.750, and a specificity of 0.646, which indicates that the model has a good value of prediction. The model predictions were in good correspondence with the actual observations, as shown by the calibration curve. The DCA results demonstrated the net clinical benefit of the nomogram model in identifying the risk of developing grade ≥3 diarrhea. The model was then externally validated. External validation ROC curve analysis showed an AUC of 0.803 (95% CI: 0.702-0.918), sensitivity of 0.784 and specificity of 0.750. The values predicted in the calibration curve agreed well with the measured values. The DCA curve showed a good net clinical benefit in the nomogram.

### Risk factor analysis and nomogram construction of grade ≥3 neutropenia

#### Univariate and multivariate analysis of grade ≥3 neutropenia

To determine the independent risk factors for the occurrence of grade ≥3 neutropenia with abemaciclib and ET, risk factors were analyzed using univariate and multivariate logistic regression as shown in **Table 3**. Univariate analysis revealed that grade ≥3 neutropenia was related to ECOG, radiotherapy in the same period/within 1-month, WBC before treatment, and NE before treatment (*p* < 0.05). To control for confounding factors, multivariate logistic regression analysis included variables with p<0.1 in the univariate analysis. The results showed that ECOG score ≥1, radiotherapy in the same period/within 1 month, and NE count ≤ 2.0 × 10^9^/L before treatment were independent risk factors for grade ≥3 neutropenia following treatment with abemaciclib and ET.

**Table 3:** Univariate and multivariate analysis of grade ≥3 neutropenia.

Variables		Univariate analysis		Multivariate analysis	
		OR (95%CI)	*P* value	OR (95%CI)	*P* value
Age	<70	Reference			
	≥70	0.685(0.318,1.477)	0.334		
Molecular typing	II	Reference			
	III	0.710(0.364,1.384)	0.314		
	IV	0.663(0.357,1.230)	0.192		
ECOG	0	Reference			
	1-2	1.754(1.096,2.809)	0.019	3.345(1.672,6.694)	0.001
	≥3	3.776(1.598,8.921)	0.002	6.059(1.892,19.402)	0.002
Molecular typing	Luminal A	Reference			
	Luminal B	1.383(0.875,2.187)	0.165		
BMI	≥25kg/m^2^	Reference			
	<25kg/m^2^	1.018(0.636,1.630)	0.940		
Menstrual status	Premenopausal/ perimenopausal period	Reference			
	After menopause	1.457(0.894,2.375)	0.131		
Metastatic site	No	Reference			
	liver	0.909(0.484,1.710)	0.768		
	bone	0.716(0.437,1.174)	0.186		
Number of Metastasis	0	Reference			
	1-2	0.713(0.450,1.128)	0.148		
	≥3	0.662(0.254,1.726)	0.662		
Previous treatments	no	Reference			
	Previous endocrine therapy	0.709(0.447,1.1230)	0.256		
	Previous chemotherapy	1.823(0.822,4.044)	0.140		
	Previous Radiotherapy	1.484(0.945,2.331)	0.086	1.461(0.705,3.027)	0.308
Underlying disease	no	Reference			
	Metabolic diseases	1.178(0.616,2.250)	0.621		
	Cardiovascular diseases	1.019(0.564,1.843)	0.949		
	Underlying liver disease	1.875(0.922,3.814)	0.083	1.153(0.466,2.855)	0.758
	Underlying gastrointestinal diseases	1.356(0.805,2.283)	0.252		
Endocrine combination	Fulvestran	Reference			
	AI	1.457(0.840,2.528)	0.180		
Anti-bone metastasis	no	Reference			
	Diphosphonate	0.657(0.357,1.209)	0.177		
	Denosumab	0.813(0.403,1.639)	0.563		
Antibiotic use > 7 days	no	Reference			
	yes	1.660(0.913,3.019)	0.097	1.856(0.749,4.600)	0.182
Simultaneous or combined radiotherapy within 1 month	no	Reference			
	yes	1.870(1.120,3.122)	0.017	2.411(1.066,5.454)	0.035
BSA		1.011(0.996,1.025)	0.148		
WBC before treatment	>6.0×10^9^/L	Reference			
	4.01-6.0×10^9^/L	0.963(0.530,1.751)	0.902		
	≤4.0×10^9^/L	4.184(2.346,7.463)	<0.001	1.499(0.592,3.793)	0.393
NE before treatment	>4.0×10^9^/L	Reference			
	2.01-4.0×10^9^/L	0.990(0.533,1.839)	0.975		
	≤2.0×10^9^/L	6.854(3.673,12.787)	<0.001	6.988(2.662,18.343)	<0.001
Other hematology before treatment	ALB	0.970(0.928,1.014)	0.178		
	ALP	0.970(0.928,1.014)	0.970		
	Cr	0.998(0.986,1.010)	0.749		
	ALT	1.003(0.987,1.019)	0.688		
	AST	0.999(0.981,1.017)	0.931		

BMI, body mass index; ET, endocrine therapy; BSA, body surface area; WBC, white blood cell count; NE, neutrophil count; ALB, albumin; ALP, alkaline phosphatase; ALT, alanine aminotransferase; AST, aspartate aminotransferase; Cr, serum creatinine.

#### Development and validation of a nomogram for grade ≥3 neutropenia

**Figure 4:**
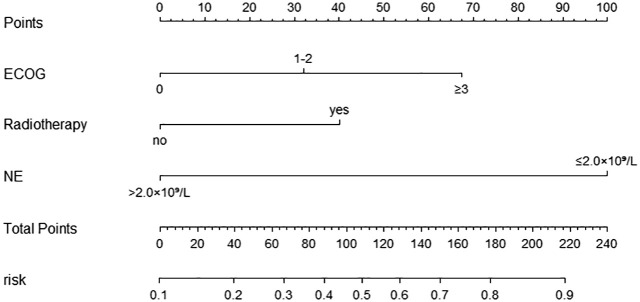
Nomogram for predicting the occurrence of ≥ grade 3 neutropenia in the modeling Group. Scores of risk factors: “ECOG” = ECOG score, 0 = 0 points, 1-2 = 32 points, ≥3 = 67 points; “Radiotherapy”= simultaneous or combined radiotherapy within 1 month, no=0 points, yes=40 points, “NE”= neutrophil level before treatment, > 2.0×10^9^/L=0 points, ≤2.0×10^9^/L=100 points.

According to the multifactorial logistic regression analysis of independent risk factors for grade ≥3 neutropenia, a nomogram model was constructed using the “rms” package in R software as shown in **Figure 4**. Scores were assigned according to the influence of the factors on the dependent variable in the nomogram, with the score line representing the estimated score for each risk factor. The incidence of grade ≥3 neutropenia was estimated based on the sum of the scores for each factor and the total score.

**Figure 5:**
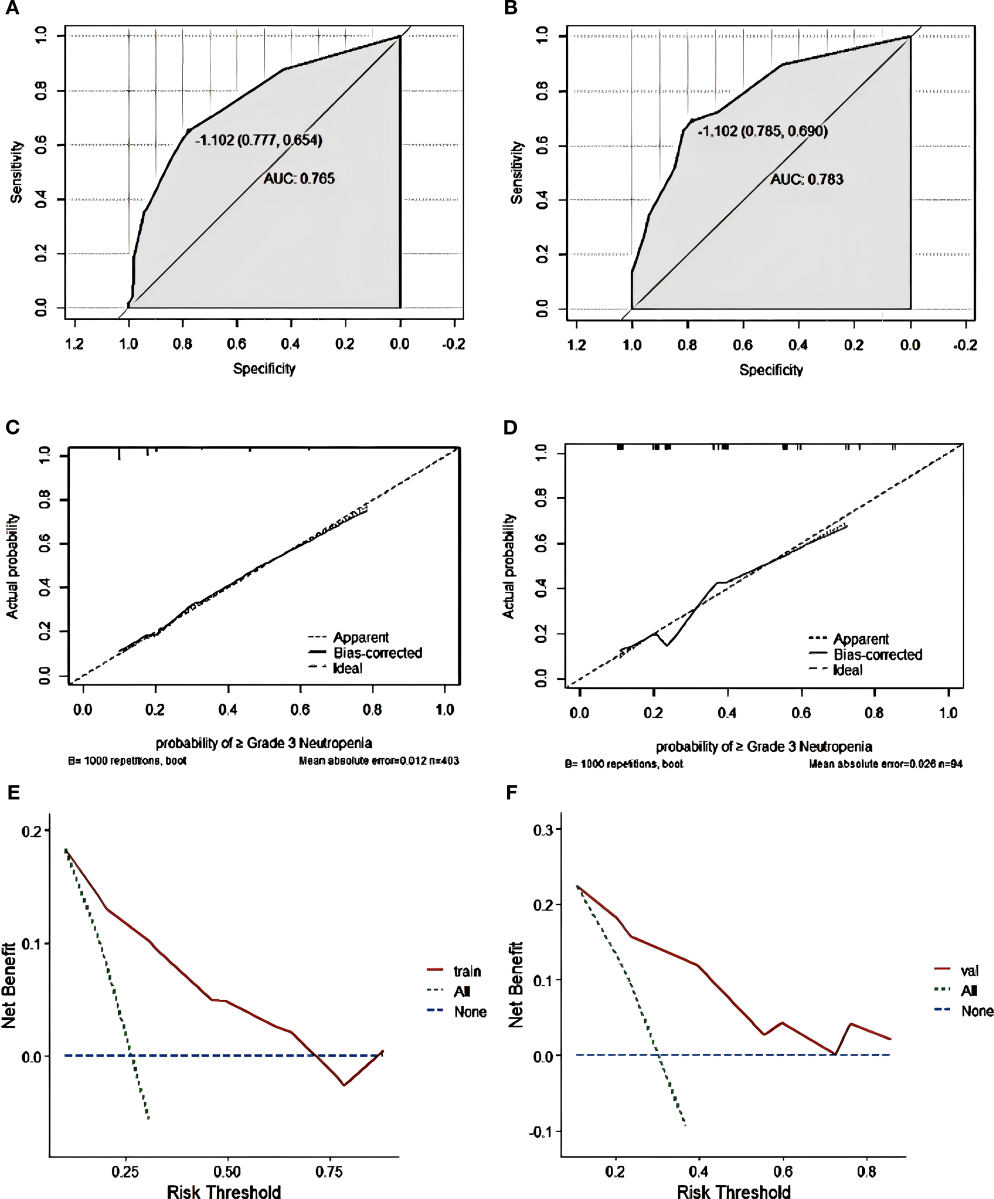
Grade ≥3 neutropenia nomogram evaluation **(A–F)**. ROC curves of the nomogram for the training set **(A)** and the external validation set **(B)**. The AUC values of the training cohort and the external validation cohort were 0.765 and 0.783, respectively. Nomogram calibration curves for training set **(C)** and validation set **(D)**. DCA curves for the training set **(E)** and validation set **(F)**.

Nomogram model performance was evaluated by C-index calculation and plotting ROC, calibration, and DCA curves as shown in **Figure 5**. The model was validated both internally and externally. In the development group, the C-index was 0.765, and the ROC curve showed that the AUC was 0.765 (95% CI: 0.711-0.818), sensitivity was 0.769, and specificity was 0.714. The ROC curve analysis of the external validation group showed that the AUC was 0.783(95% CI: 0.691-0.892), sensitivity was 0.785, and specificity was 0.690, indicating that the predictive value of the model was good. In both the development and external validation groups, the predicted values on the calibration curve were in good agreement with the measured values. The DCA results showed that both groups were clinically effective in identifying the risk of grade ≥3 neutropenia with a nomogram model within the threshold range.

## Discussion

CDK4/6 inhibitors combined with ET have become the primary recommended regimen for salvage treatment of HR+/HER2- advanced breast cancer and adjuvant intensive treatment of locally advanced breast cancer with high-risk factors. Although the focus is on anticancer efficacy in clinical practice, monitoring and managing AEs is equally important. Notably, both single severe AEs and multiple superimposed AEs place a serious burden on the physical and mental health of patients and may even necessitate treatment interruption, thereby changing the survival outcome of patients. Both the clinical trial data reported in the current study and our real-world follow-up data show that diarrhea and neutropenia are the most common adverse events leading to treatment discontinuation and dose reduction of the CDK4/6 inhibitor abemaciclib plus endocrine therapy. Our study also revealed that approximately 54.7% (272 cases) of patients had grade 3 ≥ AEs, approximately 23.1% (115 cases) of patients discontinued treatment due to intolerable AEs, and approximately 18.3% (91 cases) of patients reduced the dose. These findings were slightly higher than the statistical data from the MONARCH 2 and MONARCH 3 clinical trials. This may also reflect the differences between clinical practice and clinical trials in the population and the standardized management of the treatment process. In the present study, we compared the occurrence of diarrhea and neutropenia following the use of the combination of abemaciclib with several ET agents and found no significant differences. Therefore, this study explored the risk factors for diarrhea and neutropenia after the use of CDK4/6 inhibitor abemaciclib combined with ET based on real-world evidence and integrated the risk factors in the regression model through R language to form a nomogram prediction model, which provided a convenient and individualized prediction tool for clinicians.

In addition to inhibiting CDK4 and CDK6, abemaciclib also has a strong inhibitory effect on CDK9 (15), which is mainly distributed in the gastrointestinal tract; therefore, abemaciclib-related AEs show a high incidence of diarrhea and fatigue. However, the precise mechanism underlying the induction of diarrhea by abemaciclib remains to be elucidated. Studies have shown that abemaciclib inhibits glycogen synthase kinase-3β (GSK3β) activity in patients with diarrhea, leading to impaired epithelial cell differentiation. Furthermore, abemaciclib-induced diarrhea also significantly downregulates the Ca^2+^/calmodulin-dependent protein kinase CAMKII (16), involved in intestinal motility, which may be associated with defecation. Previous studies have indicated that the incidence of diarrhea grade 2–3 diarrhea is significantly elevated in patients aged ≥70 years, those receiving multiple drugs concurrently, and those with pre-existing gastrointestinal diseases (13, 17). Our study results also showed that age ≥70 years and underlying gastrointestinal disease were independent risk factors for grade ≥3 diarrhea. The risk of multiple AEs increases with the use of CDK4/6 inhibitors in elderly patients, and a slightly higher rate of dose adjustment and discontinuation is observed in this population (12). Toxicity and efficacy analyses have shown that older women receiving CDK4/6 inhibitors experience more severe toxicity than younger women (18, 19). The underlying causes may include organ dysfunction, comorbidities, prolonged multidrug co-administration, drug distribution, and pharmacokinetic alterations in elderly patients (11). Patients with underlying gastrointestinal diseases have diminished gastrointestinal function, rendering them susceptible to severe gastrointestinal toxicity when treated with CDK4/6 inhibitors. Therefore, other CDK4/6 inhibitors are recommended for patients with poor gastrointestinal function. In addition, we considered the influence of concurrent use of other chronic drugs on the toxicity of CDK4/6 inhibitors. A statistical analysis revealed that approximately 30–50% of prescription drugs for chronic diseases in our country are not taken as prescribed, and that drugs taken in combination by each patient are inconsistent. Our study counted the number of drugs regularly taken by patients while taking abemaciclib and found no correlation with diarrhea. However, this issue may be regarded as a limitation of our study because our numerical definitions of multiple medications did not consider the role of the respective medications, making it difficult to assess treatment safety and applicability.

Unlike chemotherapy, which inhibits the bone marrow by directly damaging naive hematopoietic cells, CDK4/6 inhibitors induce cell cycle arrest by reducing hematopoietic stem cell proliferation, allowing proliferation to be restored after dose reduction or CDK4/6 blockade (20). The incidence of neutropenia was lower with abemaciclib than with other CDK4/6 inhibitors; however, it was still one of the most frequently reported serious (grade ≥3) AEs with abemaciclib (21). In our study, ECOG score >1, radiotherapy in the same period/within 1 month, and pretreatment neutrophils ≤2.0 × 10^9^/L were identified as independent risk factors for grade ≥3 neutropenia with abemaciclib. Baseline myelosuppression with multiple CDK4/6 inhibitors has been shown in several previous studies to be an independent predictor of grade 3 or 4 neutropenia (22–24). In addition, race, ECOG score, and drug concentration have been reported to be associated with abemaciclib-induced neutropenia (13, 25). In our study, we explored the effect of baseline WBC and NE levels on the occurrence of neutropenia during the 21 days prior to treatment and analyzed them by numerical stratification (WBC: >6.0 vs. 4.01–6.0 vs. ≤4.0 × 10^9^/L; NE: >4.0 vs. 2.01–4.0 vs. ≤2.0 × 10^9^/L). The univariate results revealed an association with neutropenia only when the WBC and NE levels were below normal. After the multivariate analysis, only NE levels of ≤2.0 × 10^9^/L before treatment were a risk factor for neutropenia, which may be due to the collinearity of WBCs and NE. However, patients with low neutrophil counts before treatment were more likely to experience severe neutropenia and were at a higher risk of infection-related complications (26, 27). However, overall, careful evaluation and correction of neutropenia before treatment, in addition to close monitoring during treatment, are essential.

The ECOG score is used to assess treatment tolerance and plays an important role in aspects such as prognosis. Patients with a high ECOG score tend to have poor tolerability to antitumor therapy and a higher incidence of AEs (28). ECOG performance status (PS) was a common risk factor for diarrhea and neutropenia in our study; a higher ECOG score predicted a higher risk. Therefore, in clinical practice, the potential benefits of CDK4/6 inhibitors in patients with relatively poor physical function cannot be completely ruled out. For these patients, clinicians should fully assess the feasibility of the treatment and choose a low initial treatment dose according to the situation. Good therapeutic effects can be achieved by implementing active and effective preventive measures. In addition, our study found that prior radiotherapy was not associated with diarrhea or neutrophilic development, but radiotherapy within the same period/within 1 month has been found to be an independent risk factor for grade ≥3 neutropenia. Evidence for combination therapy with CDK4/6 inhibitors and radiotherapy is currently mostly derived from limited retrospective studies, and there are no consensus guidelines to guide practice. Given their influence on the cell cycle, these two factors may have a synergistic effect, enhancing the efficacy and toxicity of radiotherapy (29, 30). Most retrospective studies have concluded that simultaneous radiotherapy may increase the incidence and severity of hematological and gastrointestinal AEs, but does not affect the delivery of systemic therapies (31–33). However, more efficacy and safety data need to be validated in prospective studies over a longer period.

Many factors may increase the risk of AEs associated with CDK4/6 inhibitors, as suggested by previous studies, including a history of radiotherapy for bone metastases, use of antibiotics, low body weight, and drug concentrations, which may increase the risk of neutropenia (20, 24, 34, 35). Furthermore, the risk of diarrhea is higher in postmenopausal patients, untreated patients, and patients who develop metastases (7), among others. These factors were included in our study; however, the results did not show a clear correlation. Although the influence of relevant independent risk factors has been reported, there is a lack of consensus on the influencing factors and predictive models that can include these factors in the analysis. In our study, two nomogram models were created for the prediction of the risk of grade ≥3 diarrhea and neutropenia. By calculation of the C-index and drawing of ROC, calibration, and DCA curves, the models were validated internally and externally. The prediction models constructed in this study included various clinical characteristics and treatment variables with strong predictive ability. Data were obtained from different hospitals for the development and external validation groups. Both the internal and external validation data demonstrated good predictive power, discrimination, and clinical utility of the predictive column line plots, which suggests that the models can be applied to different patient populations and medical centers.

This study had several limitations. First, it was a retrospective study, which is inevitably subject to bias; therefore, the findings need to be validated by multicenter, large prospective studies in the future. In addition, owing to drug accessibility problems, we examined only one type of CDK4/6 inhibitor. Future studies could build on this study by further increasing the number of samples and investigating the risk factors associated with the use of other CDK4/6 inhibitors to improve model stability and extrapolation.

## Conclusion

This study evaluated the risk factors for grade ≥3 diarrhea and neutropenia in patients with HR+/HER2- breast cancer receiving abemaciclib in combination with ET in a real-world clinical setting. Two nomograms were generated and internally and externally validated, demonstrating that the models had good prediction performance and could be applied to various populations and medical centers. We expect this study to provide a scientific basis for the safety assessment of abemaciclib combined with ET in patients with HR+/HER2- breast cancer. Further prospective validation is required.
