# Supplementary material for: Development and validation of nomograms for predicting grade ≥3 diarrhea and neutropenia after abemaciclib combined with endocrine therapy for breast cancer: a multicenter retrospective real-world study
Source: Front Oncol. 2025 Oct 6;15:1515420. doi: 10.3389/fonc.2025.1515420 (PMC12535899; doi:10.3389/fonc.2025.1515420)
Supplement: Supplementary file 1 [file Table1.docx]

Table S1 Real-world occurrence of AEs

| Item | Any grade  [case (%)] | grade ≥3 AEs  [case (%)] | Causing discontinuation [case (%)] | Causing dose reduction  [case (%)] |
| --- | --- | --- | --- | --- |
| Diarrhea | 307（84.5%） | 72（19.8%） | 28（7.7%） | 22（6.1%） |
| Neutropenia | 233（64.2%） | 99（27.3%） | 21（5.7%） | 11（3.0%） |
| Fatigue | 187（51.5%） | 37（10.2%） | 7（1.9%） | 8（2.2%） |
| Leukopenia | 167（46.0%） | 32（8.8%） | 6（1.7%） | 4（1.1%） |
| Decreased appetite | 117（32.2%） |  | 7（1.9%） | 4（1.1%） |
| Elevated transaminases | 93（25.6%） | 7（1.9%） | 3（0.8%） |  |
| Nausea | 86（23.7%） | 4（1.1%） |  |  |
| Elevated blood creatinine | 83（22.9%） | 5（1.4%） | 4（1.1%） |  |
| Anemia | 81（22.3%） | 12（3.3%） |  |  |
| Abdominal pain | 78（21.5%） | 2（0.5%） |  |  |
| Rash | 69（19.0%） | 3（0.8%） | 5（1.4%） |  |
| Weight loss | 65（17.9%） |  |  | 3（0.8%） |
| Dizziness and headache | 63（17.3%） |  |  |  |
| Thrombocytopenia | 62（17.1%） | 5（1.4%） | 4（1.1%） |  |
| Joint pain | 53（15.7%） |  |  |  |
| Pruritus | 50（13.8%） |  |  |  |
| Infection | 50（13.8%） |  |  |  |
| Hair loss | 48（13.2%） |  |  |  |
| Numbness of limbs | 46（12.7%） |  |  |  |
| Fever | 43（11.8%） |  |  |  |
| Taste disturbance | 40（11.0%） |  |  |  |
| Prolonged QT interval | 37（10.1%） |  |  |  |
| Stomatitis | 36（10.0%） |  |  |  |

Table S2 Comparison of the incidence of diarrhea and neutropenia in different ET combinations

|  | Diarrhea of any grade | | grade ≥3 diarrhea | | Neutropenia of any grade | | grade ≥3 Neutropenia | |
| --- | --- | --- | --- | --- | --- | --- | --- | --- |
|  | yes | no | yes | no | yes | no | yes | no |
| Fulvestrant  (n=210) | 178 | 32 | 44 | 166 | 140 | 70 | 54 | 156 |
| Exemestane  (n=47) | 41 | 6 | 9 | 38 | 29 | 18 | 14 | 33 |
| Anastrozole  (n=182) | 158 | 24 | 34 | 148 | 121 | 61 | 52 | 130 |
| Letrozole  （n=58) | 48 | 10 | 12 | 46 | 30 | 28 | 16 | 44 |
| *P* | 0.847 | | 0.949 | | 0.170 | | 0.903 | |
